# Sensing of endogenous retroviruses-derived RNA by ZBP1 triggers PANoptosis in DNA damage and contributes to toxic side effects of chemotherapy

**DOI:** 10.1038/s41419-024-07175-7

**Published:** 2024-10-27

**Authors:** Fang Wang, Kaiying Li, Wensheng Wang, Hui Jiang, Jiangping He, Jin Cai, Wenqing Ren, Yaxing Zhao, Qianqian Song, Yuan He, Yanlei Ma, Xiaona Feng, Yue Liu, Jianqiang Yu, Siriporn Jitkaew, Dan Ma, Zhenyu Cai

**Affiliations:** 1 Tongji University Cancer Center, Shanghai Tenth People’s Hospital, School of Medicine, Tongji University, 200072 Shanghai, China; 2 Department of Biochemistry and Molecular Biology, School of Medicine, Tongji University, 200331 Shanghai, China; 3 Department of General Surgery, Xinqiao Hospital, Third Military Medical University, 400037 Chongqing, China; 4 Guangzhou National Laboratory, 510005 Guangzhou, China; 5 Department of Colorectal Surgery, Fudan University Shanghai Cancer Center, 200032 Shanghai, China; 6 College of Pharmacy, Ningxia Medical University, Yinchuan, 750004 Ningxia Hui Autonomous Region Yinchuan, China; 7 Center of Excellence for Cancer and Inflammation, Department of Clinical Chemistry, Faculty of Allied Health Sciences, Chulalongkorn University, Bangkok, 10330 Thailand; 8 State Key Laboratory of Cardiology and Medical Innovation Center, Shanghai East Hospital, School of Medicine, Tongji University, 200120 Shanghai, China

**Keywords:** Apoptosis, Necroptosis, Chemotherapy

## Abstract

Excessive DNA damage triggers various types of programmed cell death (PCD), yet the regulatory mechanism of DNA damage-induced cell death is not fully understood. Here, we report that PANoptosis, a coordinated PCD pathway, including pyroptosis, apoptosis and necroptosis, is activated by DNA damage. The Z-DNA binding protein 1 (ZBP1) is the apical sensor of PANoptosis and essential for PANoptosome assembly in response to DNA damage. We find endogenous retroviruses (ERVs) are activated by DNA damage and act as ligands for ZBP1 to trigger PANoptosis. By using ZBP1 knock-out and knock-in mice disrupting ZBP1 nucleic acid-binding activity, we demonstrate that ZBP1-mediated PANoptosis contributes to the toxic effects of chemotherapeutic drugs, which is dependent on ZBP1 nucleic acid-binding activity. We found that ZBP1 expression is downregulated in tumor tissue. Furthermore, in colorectal cancer patients, dsRNA is induced by chemotherapy and sensed by ZBP1 in normal colonic tissues, suggesting ZBP1-mediated PANoptosis is activated by chemotherapy in normal tissues. Our findings indicate that ZBP1-mediated PANoptosis is activated by DNA damage and contributes to the toxic side effects of DNA-damage-based chemotherapy. These data suggest that ZBP1 could be a promising therapeutic target to alleviate chemotherapy-related side effects.

## Introduction

Triggering DNA damage-induced cell death has been implicated as an important mechanism in cancer treatments, such as chemotherapy or radiotherapy [1]. However, many aspects of DNA damage-induced cell death are not fully understood. For example, theoretically, the main role of DNA damage compounds in chemotherapy is to target rapidly dividing cancer cells to trigger cell death while minimizing damage to normal, healthy cells [2]. However, normal cells can also be damaged by chemotherapy drugs, which could contribute to side effects of chemotherapy [3]. The toxic side effects of chemotherapy on the human body are not only the major dose-limiting factors in clinical practice but also cause serious reduction in the quality of life. Therefore, the identification of key mechanisms that are involved in chemotherapy-induced damage in normal cells could provide an exciting opportunity to optimize chemotherapy treatment and improve cancer care.

PANoptosis is a coordinated cell death pathway that integrates key components of pyroptosis, apoptosis and necroptosis [4]. It is regulated by a single cell death-inducing complex named PANoptosome, which contains key molecules crucial for pyroptosis, apoptosis and necroptosis and is able to activate all three to execute proinflammatory cell death [5]. Currently, several types of PANoptosome complexes have been characterized, including ZBP1- (Z-DNA binding protein 1 or DAI, DNA-dependent activator of interferon regulatory factors), AIM2- (absent in melanoma-2), RIPK1- (receptor-interacting protein kinase 1), and NLRP12 (NOD-like receptor family, pyrin domain-containing 12)-PANoptosome [6]. In ZBP1-PANoptosome, ZBP1 acts as a PANoptosis senor to activate NLRP3 inflammasome, caspase-8, RIPK1, and RIPK3 in response to influenza A virus (IAV) infection [6]. In this scenario, caspase-6 (Casp-6) was reported to facilitate the interaction between ZBP1 and RIPK3 [7]. ZBP1-mediated PANoptosis has been shown to play a critical role in infectious and inflammatory diseases such as viruses and fungi infections and septic inflammation [7–11]. However, whether ZBP1-mediated PANoptosis is involved in DNA damage-induced cell death is not investigated.

ZBP1 is a cytosolic nucleic acid sensor that plays a crucial role in the regulation of cell death and inflammation, particularly in the context of viral infection and immune responses [12, 13]. ZBP1 contains two Zα domains (Zα1 and Zα2) in its N-terminus and two RHIM (receptor-interacting protein [RIP] homotypic interaction motif) domains in the middle. ZBP1 uses its two Zα domains to bind with left-handed Z-DNA or RNA and then drives the RHIM-mediated interactions with receptor-interacting protein kinase 3 (RIPK3) to trigger inflammatory cell death [9, 14–17]. In sterile conditions, ZBP1 has been shown to recognize ERV-derived dsRNA to induce necroptosis and inflammation [18–20].

In this study, we find that ZBP1-mediated PANoptosis is activated by DNA-damaging compounds. Mechanistically, ERVs are reactivated by DNA damage and act as ligands of ZBP1 to trigger PANoptosis. We further show that ZBP1-mediated PANoptosis contributes to the toxic effects of chemotherapeutic drugs. These data suggest that ZBP1 could be a potential therapeutic target to alleviate chemotherapy-induced side effects.

## Materials and methods

### Chemicals

Etoposide (MCE, China, Cat#HY-13629), MNNG (MCE, China, Cat#HY-128612), CDDP (MCE, China, Cat#HY-17394), 5-FU (MCE, China, Cat#HY-90006), CBL0137 (MCE, China, Cat#HY-18935A) and PFN-α (MCE, China, Cat# HY-123076).

### Antibodies

#### WB

The following antibodies were from commercial sources: anti-Cleaved Caspase −3 (CST, USA, Cat#9664, 1:1000); anti-Caspase-3 (ABclonal, China, Cat#A2156, 1:1000); anti-phospho-MLKL (Abcam, USA, Cat#ab196436, 1:1000); anti-MLKL (ABclonal, China, Cat#A5579, 1:1000); anti-FLAG-Tag (ABclonal, China, Cat#AE004, 1:1000); anti-ZBP1 (Adipogen, Switzerland, Cat#AG-20B-0010-C100, 1:1000); anti-GSDME-N-terminal (Abcam, USA, Cat#ab215191, 1:1000); anti-phospho-RIPK1 (CST, USA, Cat#31122, 1:1000); anti-RIPK1 (BD, USA, Cat# 610458, 1:1000); anti-phospho-RIPK3 (CST, USA, Cat#91702, 1:1000); anti-RIPK3 (CST, USA, Cat#95702, 1:1000); anti-Caspase-6 (CST, USA, Cat#9762, 1:1000); anti-Cleaved Caspase-6 (CST, USA, Cat#9761T, 1:1000); anti-γ-H2AX (CST, USA, Cat#9718, 1:1000); anti-H2AX (ABclonal, China, Cat#A11463, 1:1000); anti-phospho-p53(CST, USA, Cat#9284, 1:1000); anti-p53 (Proteintech, China, Cat# No. 10442-1-AP, 1:1000) and GAPDH (HUABIO, China, Cat# ET1601-4, 1:100000).

#### IF/IHC

The following antibodies were from commercial sources: anti-dsRNA J2 (SCICONS, Hungary, Cat#10010200, 1:200); anti-Z-DNA/Z-RNA [Z22] (Absolute antibody, UK, Cat#T2028A11, 1:200); anti-DDDDK-Tag (ABclonal, China, Cat#AE004, 1:200); anti-ZBP1(Proteintech, China, Cat#No. 13285-1-AP, 1:100) and anti-CD45 (Invitrogen, USA, Cat# 11-0451-85, 1:100).

### Plasmids and lentiviral particles

The mammalian cell expression plasmids of Flag-tagged mouse ZBP1 and Flag-tagged mouse ZBP1-mutZα1α2 (N46A/Y50A/N122A/Y126A) were purchased from Synbio Technologies (Suzhou, China). For generating the knockout cell lines by the CRISPR-Cas9 approach, gRNA sequences were firstly cloned into pLenti-CRISPRv2 vector. The gRNA targeting sequences are as follows:

sgRIPK3 #1 sense: 5′-CTCTGGGTCCAAGTACGCTA-3′

sgRIPK3 #1 antisense: 5′-TAGCGTACTTGGACCCAGAG-3′

sgRIPK3 #2 sense: 5′-GCTCTGGGTCCAAGTACGCT-3′

sgRIPK3 #2 antisense: 5′-AGCGTACTTGGACCCAGAGC-3′

sgCasp-6 #1 sense: 5′-CAGGTTGTCTCTGTCTGCGT-3′

sgCasp-6 #1 antisense: 5′-ACGCAGACAGAGACAACCTG-3′

sgCasp-6 #2 sense: 5′-CGTTGGTGCCCCGCCTCTCT-3′

sgCasp-6 #2 antisense: 5′-AGAGAGGCGGGGCACCAACG-3′

For generating lentivirus, HEK293T cells were co-transfected with pCMV-VSV-G and pCMV-dr8.2-dvpr and either empty or sgRNA-targeted plasmids. After 24 h, supernatant was collected, and this lentiviral preparation was used to infect cells. After 24 h of infection, cells were selected with puromycin for a further 48 h.

### Cell culture and transfection

All the cell lines used in this study were obtained from the American Type Culture Collection (ATCC, Manassas, VA). Wild-type (WT), ZBP1-deficient and Zα1α2-mutated mouse dermal fibroblast (MDF) cells were isolated from postnatal day 2 of C57BL/6 mouse skin. All cells were maintained in DMEM culture media supplemented with 10% FBS (v/v), 2 mM of l‐glutamine and 100 U/ml of penicillin/streptomycin. Cells were grown at 37 °C in a humidified atmosphere with 5% CO_2_ and were harvested in all experiments from exponentially growing cultures.

The plasmids were transfected with Lipo293F™ transfection reagent (Beyotime, China) according to the manufacturer’s protocol. After 24 h, the cell lysates were analyzed by immunoblotting.

### Cell treatment and cell death assays

Cells were treated with ETO (50 μM), MNNG (0.5 mM), CDDP (50 μM) or 5-FU (50 μg/mL) for the indicated time to induce cell death. If there are any differences, a detailed description will be given in the Figure legend. Cell death was examined by propidium iodide (PI) staining. Briefly, cells were trypsinized, collected by centrifugation, washed once with PBS buffer and then resuspended in PBS containing 5 μg/mL of PI. The stained cells were subjected to flow cytometry using a BD FACS Aria II (BD Biosciences, USA). The proportions of PI‐positive cells were quantified with FlowJo™ Software (BD Biosciences, USA).

### Immunoblotting

Cell lysates were prepared for immunoblotting analysis using RIPA buffer supplemented with protease/phosphatase inhibitors and PMSF (MCE, Shanghai, China). The RIPA buffer consisted of 10 mM Tris–HCl [pH 8.0], 1 mM EDTA, 0.5 mM EGTA, 140 mM NaCl, 0.1% SDS, 1% Triton X-100, 50 mM NaF, 40 mM glycerophosphate, and 0.1 mM sodium vanadate. Cell lysates were separated by SDS–PAGE and analyzed by immunoblotting. The proteins were visualized by enhanced chemiluminescence according to the manufacturer’s instructions (Tanon, China).

### Immunoprecipitation

Cells were harvested and lysed in immunoprecipitation lysis buffer (Beyotime, China, Cat#P0013) with protease/phosphatase inhibitors and PMSF. After centrifugation, the cell lysate supernatants were immunoprecipitated with anti-FLAG magnetic beads (15 μL) (MCE, China, Cat#HY-K0207) at 4 °C overnight with rotation. Following extensive washing with lysis buffer, the proteins bound to magnetic beads were eluted by boiling in 1×SDS sample buffer (30 μL). Finally, the precipitated proteins were subjected to immunoblotting using the specified antibodies. Image J (NIH, USA) software was used for greyscale analysis of western blotting. For DNase I or RNase A treatment, cell lysates were incubated with 25 U/mL DNase I (NEB, USA, Cat#M0303S) or 1 mg/mL RNase A (MCE, China, Cat# HY-129046A) for 1 h at 4 °C. Subsequent immunoprecipitation and western blotting were then performed.

### Immunofluorescence microscopy

For tissue staining, paraffin sections of tissues were baked at 65 °C for 1 h, and then deparaffinized. Antigen retrieval was performed by boiling the sections in Citrate Antigen Retrieval Solution (Beyotime, China, Cat#P0081) for 20 min. For cell staining, cells were plated on 24-well glass slides (JingAnBiological, China, Cat#J24001), and cultured for 24 h before use in experiments. Following ETO (50 μM) or CBL0137 (5 μM) treatment, cells were fixed in 4% paraformaldehyde for 15 min and permeabilized with 0.1% Triton-X100 for 5 min. To detect dsRNA and Z-DNA in cells, cells were subjected to proteinase K treatment (0.008 U/mL) for 20–40 min at 37 °C post-fixation. If required, the cells will then be treated with RNase A (1 mg/mL), RNase III (1 mg/mL) or DNase I (25 U/ml) for 1 h at 37 °C. Subsequently, cells or tissue sections were blocked with 5% BSA-V (Solarbio, China, Cat#A8020) in PBS, and then incubated overnight with primary antibodies at 4 °C. After washing with PBS three times, slides were incubated with fluorophore-conjugated secondary antibodies for 1 h at room temperature and stained with DAPI for 5 min. Following an additional three washes in PBS, slides were mounted in ProLong Gold antifade reagent (Thermo Fisher Scientific). The staining images were acquired using the confocal laser scanning microscope (Nikon, Japan). For Laser confocal microscopy analysis, five random images (based on the total counted cell number)were captured for co-localization analysis.

### RNA interference

Three sets of small interfering RNAs (siRNAs) corresponding to nucleotide sequences of mouse p53 were synthesized and purchased from Synbio Technologies (Suzhou, China). The RNAi sequences for the primer sets were as follows:

siRNA #1 sense: 5′-CACUACAAGUACAUGUGUAdTdT-3′

siRNA #1 antisense: 5′-UACACAUGUACUUGUAGUGdTdT-3′

siRNA #2 sense: 5′-GUAAACGCUUCGAGAUGUUdTdT-3′

siRNA #2 antisense: 5′-AACAUCUCGAAGCGUUUACdTdT-3′

siRNA #3 sense: 5′-CUAUCUUGGGCCCUCAUAGdTdT-3′

siRNA #3 antisense: 5′-CUAUGAGGGCCCAAGAUAGdTdT-3′

The negative control duplex was also obtained from Synbio Technologies. The siRNA duplexes at the final concentration of 10 nM were transfected into MDF cells, using the HiPerFect Transfection Reagent (Qiagen, Germany), according to the manufacturer’s protocols. At 48 h after the siRNA transfection, the cell lysates were subjected to immunoblot analysis to determine the expression of p53.

### Proximity Ligation Assay

The Proximity Ligation Assay (PLA) was performed using the Duolink® In Situ Red Starter Kit (Sigma, USA, Cat#DUO92101) according to the manufacturer’s instructions. Briefly, cells were cultured on confocal culture dishes and then fixed in 4% paraformaldehyde (PFA) for 15 min, washed three times, permeabilized with 0.1% Triton X-100 for 5 min, and then blocked with Duolink blocking solution for 1 h at 37 °C. Cells then incubate overnight at 4 °C with primary antibodies diluted in Duolink antibody diluent. Subsequently, secondary antibodies were applied, followed by ligation and signal amplification steps according to the manufacturer’s instructions. The PLA dots were visualized using a Nikon confocal microscope.

### Real-time (RT) PCR

To measure relative gene expression by RT-PCR, total cellular RNA was isolated using TRIzol reagent (ThermoFisher, USA). The extracted RNA was reversely transcribed into cDNA using the Strand cDNA Synthesis kit (Transgen, China, Cat#AH321-01) according to the manufacturer’s instructions. Levels of mRNA encoding for RLTR45-int, RLTR1B-int or IL-6 were measured by real-time PCR using PerfectStart® Green qPCR SuperMix (Universal Passive Reference Dye) (Transgen, China, Cat#AQ602-01) in the QuantStudio 7 Flex Real-Time PCR system (ThermoFisher, USA). The ratio for the mRNA was normalized to GAPDH. All reagents, buffers and containers used for RNA work were RNase-free grade, to eliminate RNase contaminants in experiments described in this section and other relevant sections. The primer sequence of RLTR45-int and RLTR1B-int have been reported previously [19]. The primer sequence is as follows:

RLTR45-int sense: 5′-CCCCGAGAAGAGGGTCAAAA-3′

RLTR45-int antisense: 5′-AGCGGGAACTTGGTGGTATC-3′

RLTR1B-int sense: 5′-AATCCACTGTCTCCTGCGTG-3′

RLTR1B-int antisense: 5′-CCCCACTCAACTCCCGATTC-3′

mIL-6 sense: 5′-CAATGGCAATTCTGATTGTATG-3′

mIL-6 antisense: 5′-AGGACTCTGGCTTTGTCTTTC-3′

mGAPDH sense: 5′-GTTGTCTCCTGCGACTTCA-3′

mGAPDH antisense: 5′-GGTGGTCCAGGGTTTCTTA-3′

### RNA immunoprecipitation (RIP)

RIP assays were performed following the instructions of the BeyoRIP™ RIP Assay Kit (Beyotime, China, Cat# P1801S). The cell lysates were mixed with magnetic beads bound to the corresponding antibodies and incubated at 4 °C overnight. RNA was purified with protease K solution and extracted by using RNA isolated Total RNA Extraction Reagent. Subsequent RT-PCR was performed using the primers for RLTR45-int and RLTR1B-int.

### Clinical samples

Tumor and matched noncancerous colonic tissues were obtained from the first surgical resection at Xinqiao Hospital. All study participants provided written informed consent and volunteered to participate in the experiment. All samples were collected with the approval of the local ethics Committee and the institutional review board of the hospital. Detailed clinic information of the CRC patients is shown in Supplementary Table II.

### Animal experiment

All animal care and experimental procedures complied with the National Institutes of Health guidelines and were approved by the animal care and use committee of Tongji University. Animal studies are reported in compliance with the ARRIVE guidelines [21]. C57BL/6 mice were purchased from SLAC ANIMAL (Shanghai, China) unless mentioned otherwise. ZBP1^−/−^ and ZBP1^mutZα1α2^ (N46A/Y50A/N122A/Y126A) mice with C57BL/6 background were generated by Bangyao Biologicals (Shanghai, China) with CRISPR-Cas9 gene editing technology. Mice were bred and housed under SPF conditions in individually ventilated cages with a 12 h light/dark cycle and stable temperature (25 °C). All mice used in the experiments were between the age of 8–10 weeks. We used the Quick Genotyping Assay Kit for Mouse Tail (Beyotime, China, Cat#D7283S) to identify the genotype of mice. The genotyping primer sequence is as follows:

ZBP1 sense: 5′-CCTAAGTGTCCCAGGCCATA-3′

ZBP1 antisense: 5′-ATTTGCCTGGTCTCCAGATG-3′

To establish mouse models of chemotherapy-induced toxicity, ETO was injected intravenously at a dose of 20 mg/kg; CDDP was injected intraperitoneally at a dose of 25 mg/kg. No statistical method was used to predetermine the sample size. Saline injection was used in the control group. No blinding was done to the group allocation. Five days after injection, the mice were sacrificed using CO_2_ inhalation. The small intestines and lungs were collected for haematoxylin and eosin (H&E) and immunohistochemistry staining. No samples or animals were excluded from the analysis in this study.

### H&E staining and pathological analysis

The small intestine and lung tissues were fixed in formalin overnight and subsequently embedded in paraffin blocks for sectioning. The tissue sections were then subjected to H&E staining, which facilitated the visualization of the nucleus and cytoplasm, respectively. The pathological images of livers were captured using an upright microscope.

The average number of surviving crypts, villi and neutrophils within the alveolar space were counted in 20 randomly selected fields from each mouse (*n* = 6 per group). Acute lung injury scores were calculated according to the previously described method [22]. Briefly, a 3-point scale was used to score the severity of the lung injury (0 = none, 1 = medium, and 2 = severe); the number of neutrophils in the alveolar and interstitial space (0 = none, 1 = 1–5, and 2 = >5), the formation of hyaline membranes (0 = none, 1 = 1, and 2 = >1), the proteinaceous debris filling the airspaces (0 = none, 1 = 1, and 2 = >1) and the extent of alveolar septal thickening (0 = <2×, 1 = 2–4×, and 2 = >4×) were graded. Each grade was then multiplied by a factor and then summed to obtain the final score for each mouse.

For spleen injury assessment, the spleens were first weighed and then homogenized. The remaining lymphocytes were counted by hemocytometer after lysing the red blood cells using an ACK (ammonium-chloride–potassium) buffer. The mice’s weights were recorded daily for a period of five days.

### Immunohistochemistry

For immunohistochemistry analysis, intestinal tissues from mice or patients were fixed in formalin for a minimum of 24 h, followed by paraffin embedding and sectioning. The sections were then deparaffinized in xylene, rehydrated, and washed with PBS. Antigen retrieval was performed by boiling the sections in Citrate Antigen Retrieval Solution (Beyotime, China, Cat#P0081) for 20 min. Subsequently, the sections were blocked with 10% normal goat serum in PBS at room temperature for 1 h and stained with dsRNA J2 antibodies (SCICONS, Hungary, Cat#10010200, 1:200) overnight. Signals were then developed using the VECTASTIN ABC Elite kit (Vector Laboratories, USA) and the DAB Substrate Kit (Vector Laboratories, USA).

Four human tissues, the staining sections were then reviewed and scored as follows: Cell with <10% staining was rated as negative staining (−, 1); cell with 10–49% staining was scored as (+, 2); cell with 50–74% staining was scored as (++, 3); cell with 75–100% staining was scored as (+++, 4). According to the score of staining intensity, 0 points for no positive staining (negative), 1 point for light yellow particles (weak positive), 2 points for brown particles (strong positive), and 3 points for brown particles (strong positive). The final score was defined as the staining number score multiplied by the staining color score [23].

### RNA-seq and TE RNA-seq analysis

Total RNA was isolated using RNeasy kit (Qiagen). Purified total RNA was quantified by Qubit (Invitrogen, USA) and analyzed by an Agilent Bioanalyzer to assess RNA integrity. rRNA-depleted RNA was generated using a NEBNext® rRNA Depletion Kit (New England Biolabs, Cat#E6310S). The directional RNA library was constructed with a NEBNext® Ultra II Directional RNA Library Prep Kit for Illumina® (New England Biolabs, Cat#E7760L) according to the manufacturer’s instructions. Library sequencing was performed by Novogene (Tianjin, China) using an Illumina HiSeq 4000 platform.

RNA-seq data processing was performed as described previously [24]. For TE analysis, the reads were mapped to the mouse genome (mm10) using the STAR aligner (v2.5.4b) [25]. For bi-directional ERV transcripts analysis, the strand-specific RNA-seq fastq data were aligned to mm10 genome using STAR, and then the forward and reverse strands were separated by samtools (version 1.17) [26] with the -f flag parameter. The counts for each gene or TE family were counted using scTE9 [24]. DESeq2 (v1.20.0) was used for data normalization and differential expression analysis [27]. Differentially expressed TEs were defined by a Benjamini–Hochberg-corrected *P* value < 0.05 and an absolute fold change > 2 (for genes).

### Statistical analysis

The Student’s *t*-test and one-way analysis of variance (ANOVA) were used for comparison among all different groups represented with the mean values ± standard errors. Log-rank (Mantel–Cox) test was performed for survival curve analysis using GraphPad Prism 8 (GraphPad Software, USA). All experiments were repeated at least three times with similar results. *P* < 0.05 was considered statistically significant.

## Results

### ZBP1 is involved in DNA damage-induced cell death

**Fig. 1:**
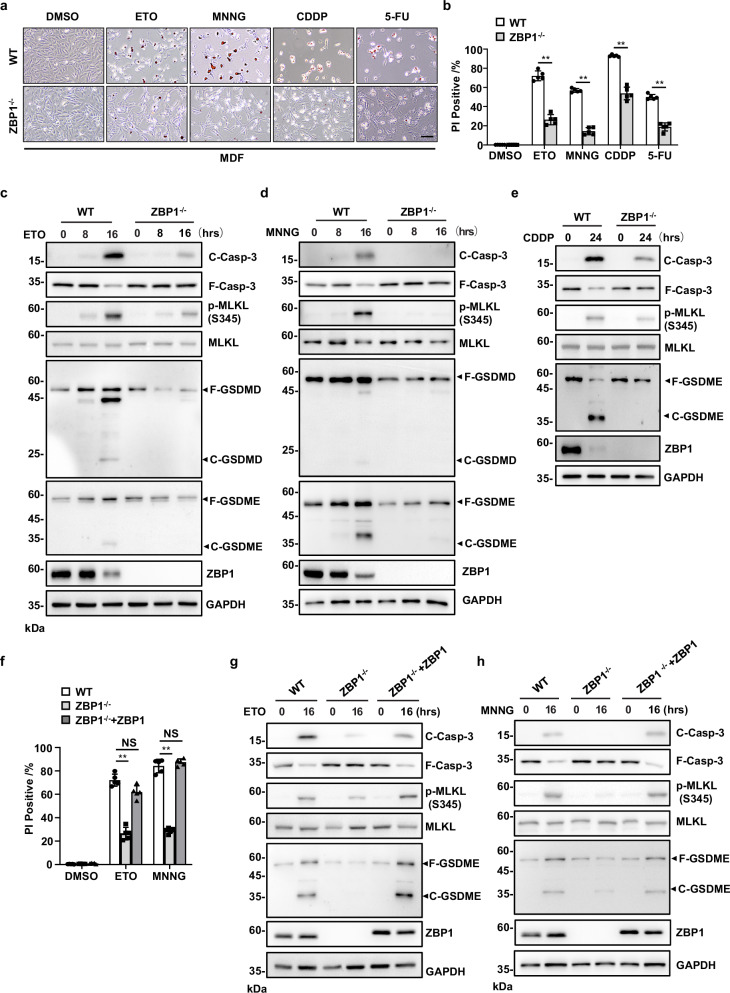
ZBP1 mediates DNA damage-induced cell death. **a** WT or ZBP1^−/−^ MDFs were treated with ETO (50 μM), MNNG (500 μM), CDDP (50 μM) or 5-FU (50 μg/mL) for 16 h and representative merged images of phase-contrast and PI staining were shown. Scale bar, 100 μm. **b** Cell death of the MDFs in (**a**) was determined by PI staining. **c** MDFs were treated with ETO, **d** MNNG or **e** CDDP at the indicated time points. Cells were lysed and immunoblotted with the indicated antibodies. hrs, hours. **f** WT, ZBP1^−/−^ or ZBP1 reconstituted MDFs were treated with ETO or MNNG for 16 h, and cell death was determined by PI staining. **g** WT, ZBP1^−/−^ or ZBP1 reconstituted MDFs were treated with ETO or **h** MNNG at the indicated time points. Cells were lysed and immunoblotted with the indicated antibodies. Data are from at least three independent experiments. Bar graphs represent the mean ± SD from three independent experiments. Statistical analysis was performed using a two-sided Student’s *t*-test. NS no significant (*P* > 0.05); *P < 0.05; **P < 0.005 (two-way ANOVA).

It is known that ZBP1 mediates various PCD pathways in infectious and sterile conditions [12]. However, whether ZBP1 is involved in DNA damage-induced cell death is unknown. To examine the role of ZBP1 in DNA damage-induced cell death, we first used wild-type (WT) and ZBP1-null (ZBP1^−/−^) mouse (Supplementary Fig. 1) dermal fibroblasts (MDFs) to compare the sensitivity of these cells to DNA damage-induced cell death. By treating the cells with different DNA-damaging compounds, including topoisomerases inhibitor etoposide (ETO), DNA alkylating agent N-methyl-N’-nitro-N-nitrosoguanidine (MNNG), DNA cross-linking agent cisplatin (CDDP) and antimetabolites inhibitor 5-FU, we found DNA damage-induced cell death was significantly attenuated in ZBP1^−/−^ MDFs compared with WT MDFs, as observed with microscopy and quantitated by propidium iodide (PI) staining (Fig. 1a and b). Since ZBP1 controls the activation of multiple types of PCD pathways, including pyroptosis, necroptosis and apoptosis [13], we then investigated whether these types of PCD were affected in ZBP1^−/−^ MDFs. By checking the commonly used markers for PCD pathways, including caspase-3 (Casp-3) cleavage for apoptosis, MLKL phosphorylation for necroptosis, and Gasdermin-D (GSDMD) or E (GSDME) cleavage for pyroptosis, we found apoptosis, necroptosis and pyroptosis were all activated by treatment with ETO, MNNG, and CDDP in WT MDFs, while the activation of these forms of PCD was suppressed in ZBP1^−/−^ MDFs (Fig. 1c-e). Furthermore, when ZBP1 expression was reconstituted in ZBP1^−/−^ MDFs, the sensitivity of these cells to ETO- and MNNG-induced cell death was restored in a comparable manner to WT MDFs (Fig. 1f). Additionally, we found Casp-3 cleavage, MLKL phosphorylation and GSDME cleavage were all reactivated by ETO- and MNNG-treatment in ZBP1-reconstituted MDFs (Fig. 1g and h). Thus, these results indicate that ZBP1 plays a critical role in DNA damage-induced PCD pathways, including pyroptosis, apoptosis and necroptosis.

### DNA damage induces PANoptosis by promoting ZBP1-PANoptosome assembly

**Fig. 2:**
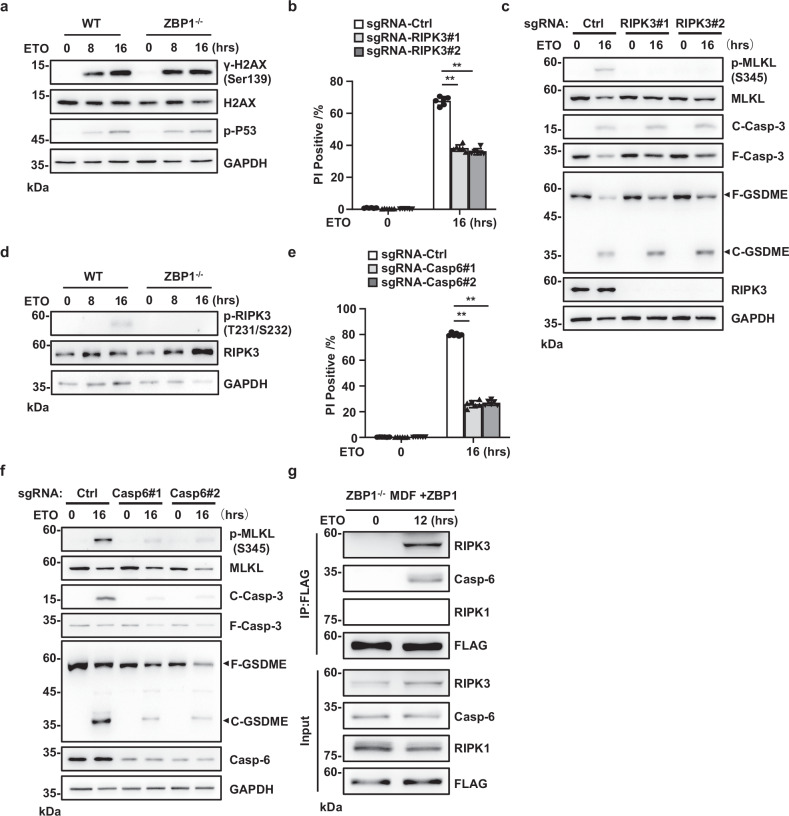
DNA damage induces PANoptosis by promoting ZBP1-PANoptosome assembly. **a** WT or ZBP1^−/−^ MDFs were treated with ETO at the indicated time points. Cells were lysed and immunoblotted with the indicated antibodies. **b** sgRNA-Control, sgRNA-RIPK3#1 and sgRNA-RIPK3#2 MDFs were treated with vehicle control or ETO for 16 h and cell death was determined by PI staining. **c** Cells in (**b**) were lysed and immunoblotted with the indicated antibodies. **d** WT or ZBP1^−/−^ MDFs were treated with ETO at the indicated time points. Cells were lysed and immunoblotted with phospho-RIPK3 and RIPK3 antibodies. **e** sgRNA-Control, sgRNA-Casp6#1 and sgRNA-Casp6#2 MDFs were treated with vehicle control or ETO for 16 h and cell death was determined by PI staining. **f** Cells in (**e**) were lysed and immunoblotted with the indicated antibodies. **g** ZBP1 reconstituted MDFs were treated with ETO for 12 h. Cell lysates were immunoprecipitated with FLAG antibody (IP: FLAG) and analyzed by immunoblotting with indicated antibodies. Data are from at least three independent experiments. Bar graphs represent the mean ± SD from three independent experiments. Statistical analysis was performed using a two-sided Student’s *t*-test. NS not significant (*P* > 0.05); *P < 0.05; **P < 0.005 (two-way ANOVA).

To investigate the action mechanism of ZBP1 in DNA damage-induced cell death, we first tested whether ZBP1 was involved in DNA damage response pathways by examining the phosphorylation of γH2AX and p53, two proteins known to be activated specifically in response to DNA damage [28]. We found that loss of ZBP1 had no effects on ETO-induced phosphorylation of γH2AX and p53, suggesting ZBP1 is not involved in DNA damage response pathways (Fig. 2a). Since DNA damage induces p53-mediated apoptosis, we then tested whether ZBP1-mediated cell death is dependent on p53 signaling or not in DNA damage. We first knocked down p53 by siRNA in MDFs and found that p53 knockdown had no effect on ETO or CDDP-induced apoptosis, necroptosis, and pyroptosis (Supplementary Fig. 2a and b). To further validate these results, we treated the cells with p53 inhibitor PFN-α, which has been shown to protect cortical neurons from ETO-induced apoptosis [29]. We found that p53 inhibitor PFN-α had no effects on ETO or CDDP-induced apoptosis, necroptosis, and pyroptosis in MDFs (Supplementary Fig. 2c and d). Therefore, we conclude that ZBP1-mediated cell death is independent of p53 signaling in MDFs.

PANoptosis is a coordinated cell death pathway that integrates key components of apoptosis, necroptosis, and pyroptosis [6]. As we found ZBP1 was required for DNA damage-induced pyroptosis, apoptosis, and necroptosis, we then investigated whether DNA damage was able to induce ZBP1-mediated PANoptosis. In the necroptosis signaling pathway, receptor-interacting serine/threonine protein kinase 3 (RIPK3) is the key upstream kinase of MLKL [30, 31]. We found CRISPR/Cas9-mediated knockout of RIPK3 partially protected MDFs against ETO-induced cell death (Fig. 2b). Additionally, knockout of RIPK3 blocked MLKL phosphorylation but had no effects on cleavage of Casp-3 and GSDME in ETO-treated cells (Fig. 2c), indicating RIPK3 is essential for DNA damage-induced necroptosis but not apoptosis and pyroptosis. Moreover, compared with WT MDFs, ETO-induced RIPK3 phosphorylation was significantly attenuated in ZBP1^−/−^ MDFs (Fig. 2d), suggesting ZBP1 acts upstream of RIPK3 to regulate DNA damage-induced necroptosis. A previous study reported that Casp-6 is required for virus-induced PANoptosis by regulating ZBP1-PANoptosome assembly [7]. To test whether Casp-6 was involved in DNA-damage-induced cell death, we first knocked out Casp-6 in MDFs and found that loss of Casp-6 protected cells against ETO-induced cell death (Fig. 2e). Additionally, compared with the control cells, ETO-induced Casp-3 cleavage, MLKL phosphorylation and GSDME cleavage were all suppressed by Casp-6 knockout (Fig. 2f), suggesting Casp-6 is required for DNA damage-induced PANoptosis.

Given that ZBP1 regulated RIPK3 phosphorylation in DNA damage and Casp-6 is required for DNA damage-induced PANoptosis, we then tested whether ZBP1 could form a PANoptosome complex with RIPK3 and Casp-6 in response to DNA damage. As ZBP1 antibody is not good for immunoprecipitation [32], we used ZBP1-reconstituted MDFs by stably expressing Flag tagged-ZBP1 protein in immortalized ZBP1^−/−^ MDFs and performed immunoprecipitation experiment with anti-Flag antibody. As shown in Fig. 2g, RIPK3 and Casp-6 protein were both co-precipitated with Flag tagged-ZBP1 after ETO treatment, indicating DNA damage induces ZBP1-PANoptosome assembly. Furthermore, although it has been shown that RIPK1 is crucial for DNA damage-induced apoptosis and necroptosis [33–35], we could not detect the presence of RIPK1 in the ZBP1–PANoptosome complex, suggesting RIPK1 is not involved in DNA damage-induced PANoptosis (Fig. 2g). Additionally, we found ZBP1 expression was decreased by ETO or CDDP treatment, while the expression of other proteins including RIPK3, Casp-6 and RIPK1 did not change during ETO or CDDP treatment (Supplementary Fig. 3). Taken together, these data indicate that DNA damage induces ZBP1-PANoptosome assembly to drive PANoptosis.

### The Zα domain of ZBP1 is required for DNA damage-induced PANoptosome assembly and PANoptosis

**Fig. 3:**
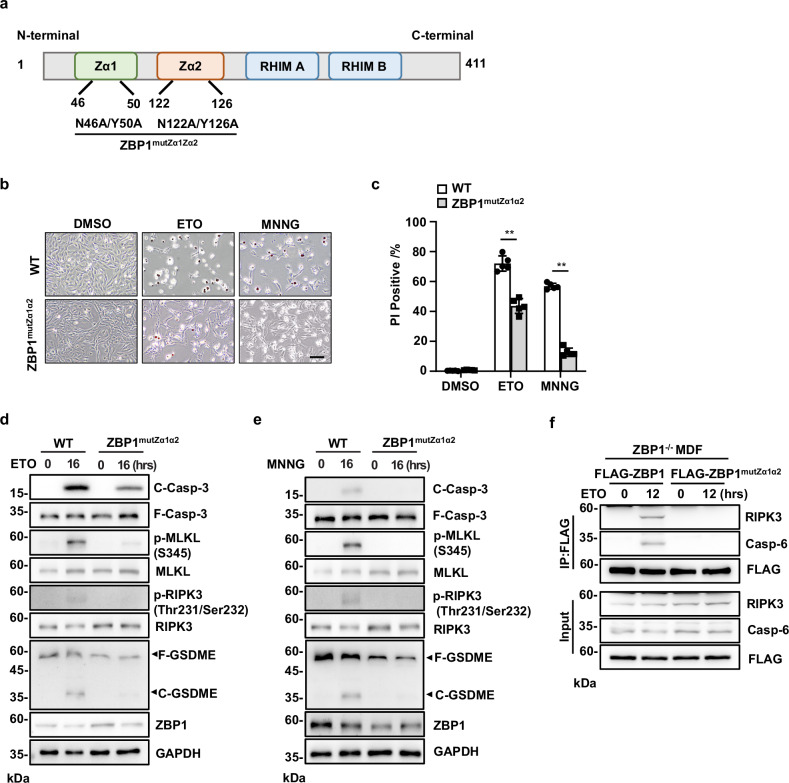
The Zα domain of ZBP1 is required for DNA damage-induced PANoptosis. **a** Schematic representation of full-length ZBP1. ZBP1 encodes two N-terminal Zα domains and two C-terminal RHIM domains. **b** WT or ZBP1^mutZα1α2^ MDFs were treated with ETO or MNNG for 16 h, and representative merged images of phase-contrast and PI staining were shown. **c** Cell death of the MDFs in (**b**) was determined by PI staining. **d** Cells in (**b**) were treated with ETO or **e** MNNG for 16 h. Cells were lysed and immunoblotted with the indicated antibodies. **f** Flag-ZBP1 or Flag-ZBP1^mutZα1α2^ were ectopically expressed in ZBP1^−/−^ MDFs and treated with ETO for 12 h, respectively. Cell lysates were immunoprecipitated with FLAG antibody (IP: FLAG) and analyzed by immunoblotting with indicated antibodies. Data are from at least three independent experiments. Bar graphs represent the mean ± SD from three independent experiments. Statistical analysis was performed using a two-sided Student’s *t*-test. NS not significant (*P* > 0.05); *P < 0.05; **P < 0.005 (two-way ANOVA).

ZBP1 protein contains two Zα domains (Zα1 and Zα2) in its N-terminus, which mediate the interactions with left-handed Z-DNA or RNA. It also contains two receptor-interacting protein homotypic interaction motif (RHIM) domains in the center part, which mediate the interactions with other RHIM domain-containing proteins such as RIPK1 and RIPK3 (Fig. 3a) [12]. To investigate how ZBP1 senses the signal to trigger PANoptosis in DNA damage, we generated a knock-in mouse with mutations at two Zα domains of ZBP1 (ZBP1^mutZα1α2^) (Supplementary Fig. 4), which results in the expression of a mutant ZBP1 that is unable to interact with Z-form nucleic acids [36]. We then isolated MDFs (MDF^mutZα1α2^) from the mice and compared the sensitivity of these cells with WT MDFs to DNA damage-induced cell death. As observed with microscopy and quantitated by PI staining, we found DNA damage-induced cell death was significantly reduced in MDFs^mutZα1α2^ compared to WT MDFs (Fig. 3b and c). Moreover, ETO- and MNNG-induced Casp-3 cleavage, MLKL phosphorylation and GSDME cleavage were all attenuated in MDF^mutZα1α2^ cells (Fig. 3d and e), indicating the Zα domains of ZBP1 are required for DNA damage-induced PANoptosis.

To investigate whether the Zα domains of ZBP1 regulate PANoptosome assembly in DNA damage, we reconstituted immortalized ZBP1^−/−^ MDFs with Flag tagged-ZBP1 containing Zα domain mutations (Flag-ZBP1-mutZα1α2) and performed immunoprecipitation experiment with anti-Flag antibody. As shown in Fig. 3f, compared with Flag-ZBP1 protein, Flag-ZBP1-mutZα1α2 protein failed to co-precipitate with RIPK3 and Casp-6 after ETO treatment, suggesting the Zα domain of ZBP1 is required for DNA damage-induced PANoptosome assembly. Therefore, these data indicate that the Zα domains of ZBP1 are required for DNA damage-induced PANoptosome assembly and PANoptosis.

### ZBP1 binds dsRNA to promote PANoptosome assembly in DNA damage

**Fig. 4:**
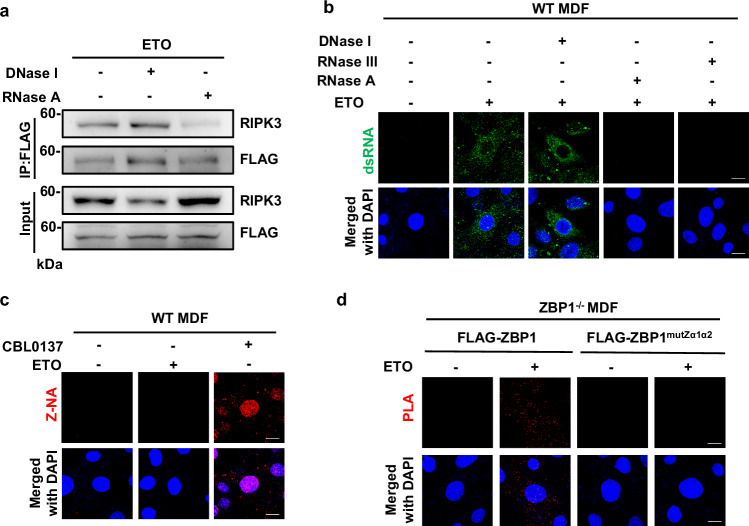
ZBP1 binds dsRNA to promote PANoptosome assembly in DNA damage. **a** ZBP1 reconstituted MDFs were treated with ETO for 12 hrs. Cells were lysed and treated with DNase I or RNase A for 1 h. Cell lysis was then immunoprecipitated with FLAG antibody (IP: FLAG) and analyzed by immunoblotting with indicated antibodies. **b** WT MDFs were treated with ETO for 12 h and then exposed to RNase III, RNase A or DNase I for 1 h, respectively. Cells were stained with anti-dsRNA J2 antibody and detected by immunofluorescence. **c** WT MDFs were treated with ETO for 12 h or CBL0137 (5 μM) for 4 h. Cells were stained with anti-Z-NA Z22 antibody and detected by immunofluorescence. **d** ZBP1-reconstituted MDFs were treated with ETO for 12 h. The physical associations between FLAG-ZBP1 and dsRNA were detected as red spots using PLA assay. Scale bar, 20 μm. Data are from at least three independent experiments.

As the Zα domains of ZBP1 mediate the binding of Z-form nucleic acids to ZBP1, we then investigated whether some nucleic acid signals were triggered by DNA damage and required for PANoptosome assembly. To determine whether ZBP1-mediated PANoptosome assembly in response to DNA damage requires DNA or RNA, we first treated the cell lysate of ZBP1-reconstituted MDFs with DNase I to degrade DNA or RNase A to degrade RNA, respectively and then performed immunoprecipitation experiment with anti-Flag antibody. We found only RNase A treatment diminished the interaction between ZBP1 and RIPK3 (Fig. 4a), suggesting RNA is required for ZBP1-PANoptosome assembly in DNA damage. We then examined if dsRNA is produced in DNA damage by performing immunofluorescent staining with the specific anti-dsRNA antibody J2. As shown in Fig. 4b, we found the signal produced by the J2 antibody was significantly increased in the cytoplasm after ETO treatment. Moreover, the signal was sensitive to RNaseA and dsRNA-specific RNase III treatment, but not to DNaseI treatment (Fig. 4b), suggesting DNA damage induces the production of dsRNA. As ZBP1 was reported to recognize Z-form nucleic acids through its Zα domains, we then examined whether ETO-induced dsRNA was Z-form by performing immunofluorescent staining with an antibody (Z22) specific for Z-form DNA or RNA. As shown in Fig. 4c, we could not detect the signal produced by Z22 antibody in ETO-treated cells, although this antibody detected Z-form DNA very well in the cells treated with CBL0137, a potent inducer of Z-form DNA in mammalian cells [37]. To examine whether DNA damage-induced dsRNA could bind to ZBP1, we performed a proximity ligation amplification assay (PLA) in ZBP1-reconstituted MDFs. Confocal imaging by using antibodies against dsRNA and Flag showed a marked increase of dsRNA-Flag-ZBP1 association in Flag-ZBP1-reconstituted MDFs, but not in Flag-ZBP1-mutZα1α2-reconstituted MDFs in response to ETO treatment (Fig. 4d). Taken together, these data suggest that dsRNA is induced by DNA damage and can be recognized by ZBP1 through its Zα domains to trigger PANoptosome assembly.

### ERVs are transcriptionally upregulated by DNA damage and act as ligands for ZBP1

**Fig. 5:**
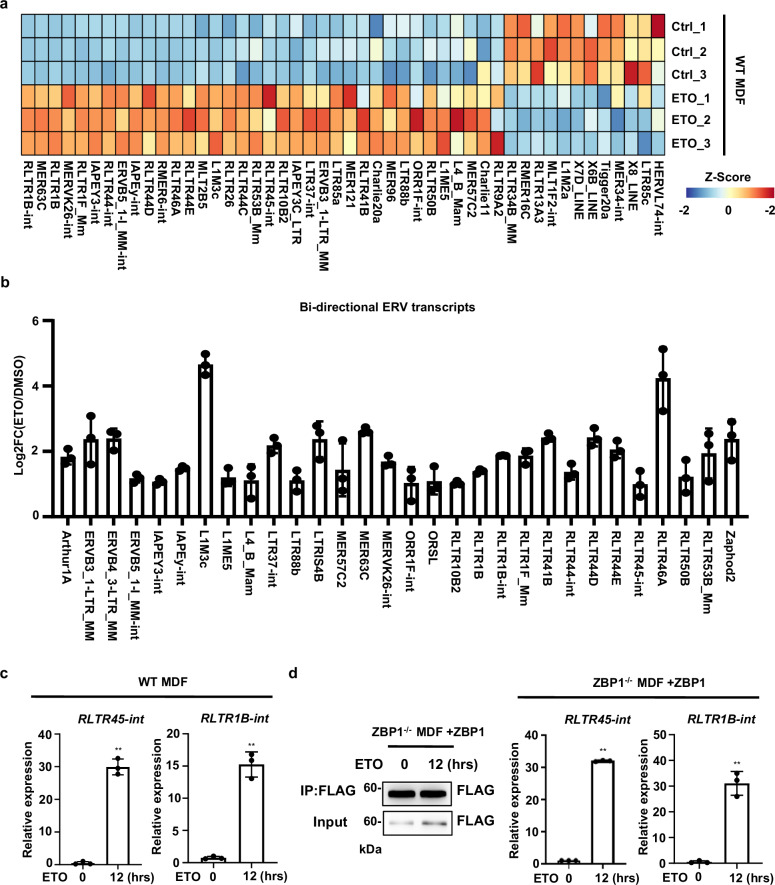
ERVs are transcriptionally upregulated by DNA damage and act as ligands for ZBP1. **a** WT MDFs were treated with ETO for 12 h. Heatmaps for differential expression of ERVs between control and ETO-treated cells were shown (FDR < 0.05). **b** Fold changes of Bi-directional ERV transcripts that are upregulated by ETO-treatment. **c** RT-PCR analysis of RLTR45-int and RLTR1B-int in WT MDF cells treated with ETO for 12 h. The RT-PCR data were normalized to GAPDH and presented as fold changes of gene expression in the test samples compared to the control. **d** ZBP1 reconstituted MDFs were treated with vehicle control or ETO for 12 h. Left, Flag-tagged ZBP1 was immunoprecipitated with FLAG antibody and analyzed by immunoblotting with FLAG antibody. Right, the co-immunoprecipitated RNA was purified to determine the expression of RLTR45-int and RLTR1B-int by real-time PCR. Data are representative of at least three independent experiments. Bar graphs represent the mean ± SD from three independent experiments. Statistical analysis was performed using a two-sided Student’s *t*-test. NS, not significant; *P < 0.05; **P< 0.005 (two-way ANOVA).

Upon ERV activation, dsRNA species may be formed by pairing ERV RNA with cellular antisense transcripts [38]. It has been well established that ZBP1 can recognize dsRNA from ERVs under sterile conditions, which is essential for ZBP1 activation [18, 19]. To test if ERV-derived dsRNA is involved in DNA damage-induced PANoptosis, we first examined whether ERV elements were transcriptionally activated by DNA damage. By examining the RNA-seq data from untreated control and ETO-treated MDFs, we identified a set of retrotransposons that were upregulated by ETO, and most of them belong to long terminal repeat (LTR)-containing ERVs (Fig. 5a and Supplementary Table I). Importantly, a number of upregulated ERVs were expressed in both sense and antisense directions with overlapping sequences, raising the probability that these ERVs could pair and form dsRNAs (Fig. 5b and Supplementary Table I). We then further confirmed that the two ERVs identified by RNA-Seq, RLTR45-int and RLTR1B-int, were upregulated by ETO or CDDP treatment through Real-time (RT)-RCR analysis (Fig. 5c, Supplementary Fig. 5a). To tested whether ERV derived-dsRNA could bind ZBP1, we performed RNA-immunoprecipitation and RT-PCR experiments with Flag antibody in ZBP1-reconstituted MDFs. As shown in Fig. 5d and Supplementary Fig. 5b, we found RLTR1B-int and RLTR45-int were markedly increased in Flag-ZBP1 precipitated samples upon ETO or CDDP-treatment, respectively. Together, these data indicate that ERVs are transcriptionally upregulated by DNA damage and can be recognized by ZBP1 in DNA damage.

### ZBP1-mediated PANoptosis contributes to chemotherapy-induced toxicity in mice

**Fig. 6:**
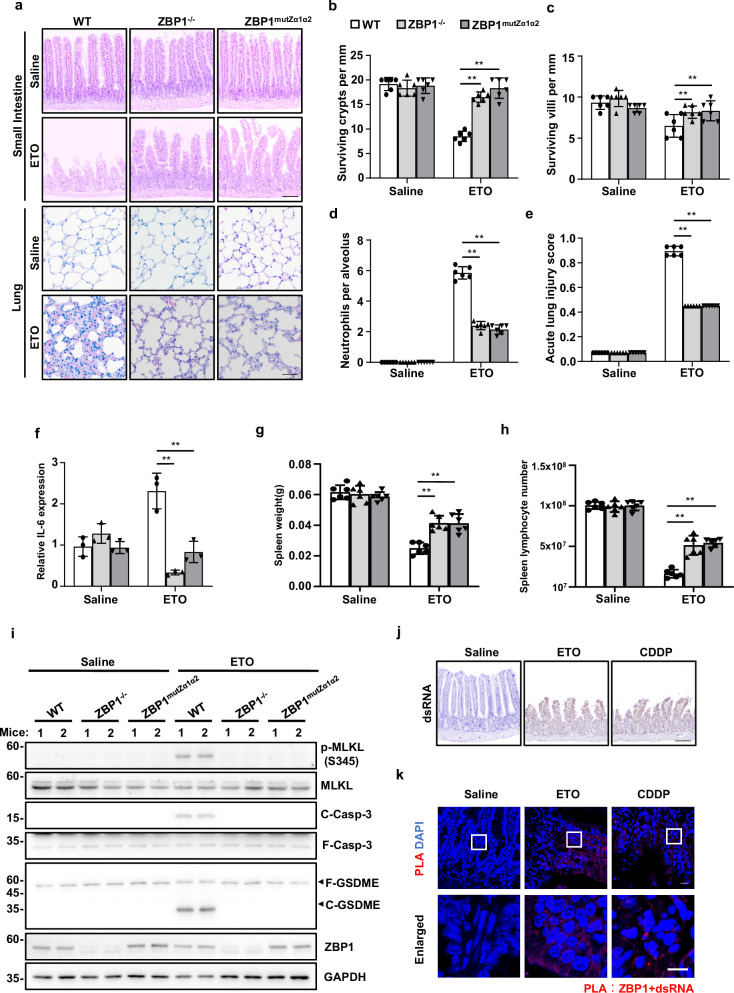
ZBP1-mediated PANoptosis contributes to chemotherapy-induced toxicity in mice. **a** WT, ZBP1^−/−^ or ZBP1^mutZα1α2^ mice were injected intravenously with ETO (20 mg/kg, *n* = 6 for each group) to establish a mice model of chemotherapy-induced toxicity. Representative H&E staining images of the small intestine and lung sections were shown. Scale bar, 100 μm. **b** Numbers of surviving crypts and **c** villi in each group of mice. **d** Numbers of neutrophils within the alveolar space in each group of mice. **e** Quantification of acute lung injury scores in each group of mice. **f** Real-time PCR analysis of IL-6 expression in lung tissue (*n* = 3 in each group of mice) was shown as mean ± SD. **g** Spleen weight and **h** total lymphocyte number were quantitated in each group of mice (*n* = 6). **i** Small intestine tissues from (**a**) were lysed and immunoblotted with the indicated antibodies. **j** Small intestine tissue sections from Saline-, ETO- or CDDP-treated mice were stained with anti-dsRNA J2 antibody. Scale bar, 100 μm. **k** Small intestine tissue sections from Saline-, ETO- or CDDP-treated mice were examined by PLA assay using anti-ZBP1 and anti-dsRNA J2 antibodies. Scale bar, 20 μm. Data are representative of at least three independent experiments. Bar graphs represent the mean ± SD from three independent experiments. Statistical analysis was performed using a two-sided Student’s *t*-test. NS not significant; *P < 0.05; **P < 0.005 (two-way ANOVA).

DNA-damaging agents including ETO, CDDP and 5-FU are among the most effective chemotherapy agents in cancer treatment, however, the clinical utility of these effective chemotherapy agents is dose limited by their toxic adverse effects [3]. Since we found DNA damage-induced ZBP1-mediated PANoptosis, we then tested whether ZBP1 contributed to the adverse effects of DNA-damaging agents used in chemotherapy. To do so, a mouse model of chemotherapy-induced toxicity was established by intravenous injection of mice with ETO. We found that ETO severely disrupted the crypts and villi in small intestines and resulted in severe vascular damage, increased neutrophil infiltration and IL-6 expression in the lung of WT mice, while these effects were significantly attenuated in ZBP1^−/−^ and ZBP1^mutZα1α2^ mice (Fig. 6a-f). Additionally, the spleen weight and the number of spleen lymphocytes were significantly reduced by ETO treatment in WT mice compared to ZBP1^−/−^ and ZBP1^mutZα1α2^ mice (Fig. 6g and h). Moreover, peritoneal injection of CDDP also caused loss of the crypts in small intestine, increased vascular damage and neutrophil infiltration in lung and decreased number of spleen lymphocytes in WT mice, and all of these effects were alleviated in ZBP1^−/−^ and ZBP1^mutZα1α2^ mice (Supplementary Fig. 6). To further confirm PANoptosis is involved in chemotherapy-induced toxicity, we then examined Casp-3 cleavage, MLKL phosphorylation and GSDME cleavage in small intestines of the mice and found the activation of these PANoptotic markers by ETO-treatment was blocked in Zbp1^−/−^ and ZBP1^mutZα1α2^ mice (Fig. 6i). Furthermore, we found ETO- and CDDP-treatment induced dsRNA production in mice small intestines (Fig. 6j). We then performed PLA assay by using J2 and ZBP1 antibodies and found ETO- or CDDP-treatment markedly increased red fluorescence signal of PLA in mice small intestines compared to vehicle control treatment, which suggests chemotherapy-induced dsRNA can be recognized by ZBP1 in vivo (Fig. 6k). Taken together, these data suggest that ZBP1-drived PANoptosis contributes to chemotherapy-induced toxicity and inflammation in mice.

### Chemotherapy-induced dsRNA is sensed by ZBP1 in noncancerous tissues from patients with colorectal cancer (CRC)

**Fig. 7:**
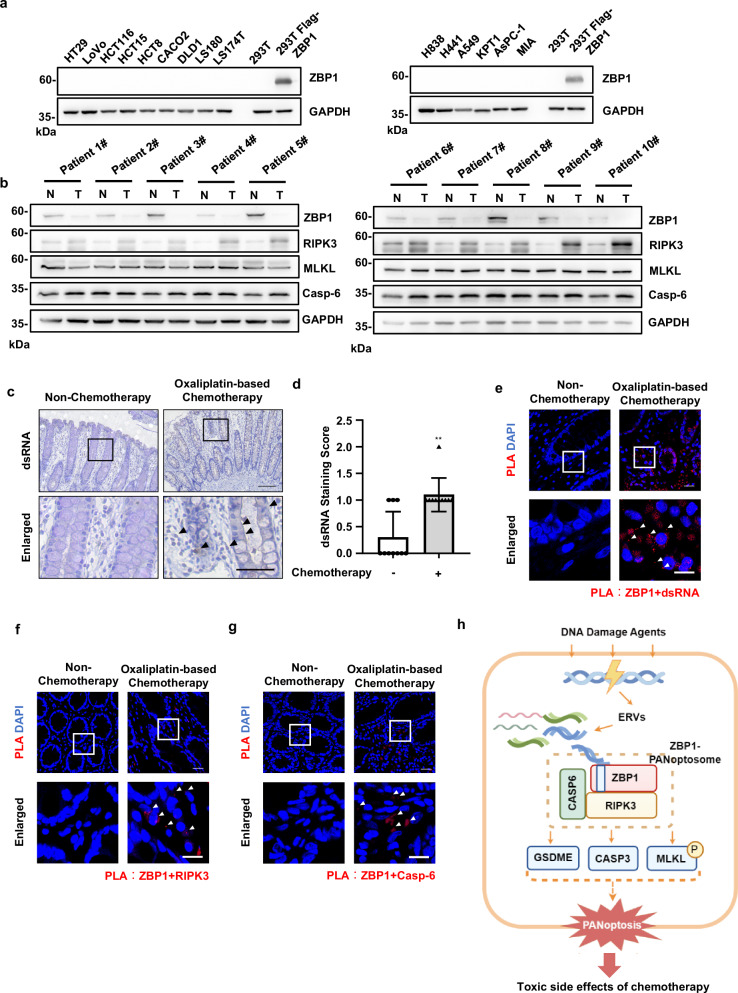
Chemotherapy-induced dsRNA is sensed by ZBP1 in noncancerous tissues from the patients with CRC. **a** Various types of human cancer cell lines were lysed and immunoblotted with anti-ZBP1 and anti-GAPDH antibodies. **b** Normal colonic and tumorous tissues from CRC patients were lysed and immunoblotted with indicated antibodies. **c** IHC analysis of dsRNA expression in normal colonic tissues from CRC patients with/out chemotherapy (*n* = 10 for each group). Scale bar, 200 μm. The complete set of images is presented in Supplementary Fig. 7. **d** Quantification of dsRNA expression level in the samples from (**c**). **e** Normal colonic tissues from (**c**) were examined by PLA assay with anti-ZBP1 antibody and anti-dsRNA J2 antibody, or **f** anti-RIPK3 antibody, or **g** anti-Casp-6 antibody. Representative images of the PLA assay were shown. Arrowheads indicate the PLA speck. **h** A working model of ZBP1-derived PANoptosis in DNA damage. ERVs are activated by DNA damage and sensed by ZBP1 to trigger RIPK3-Casp6-mediated PANoptosome assembly and PANoptosis. The activation of ZBP1-derived PANoptosis further contributes to chemotherapy-induced toxicity. Bar graphs represent the mean ± SD from three independent experiments. Statistical analysis was performed using a two-sided student’s t-test. NS, not significant; **p < 0.005 (two-way ANOVA).

Expression of ZBP1 has been shown to be downregulated in tumor tissues [39]. Consistent with this study, we found ZBP1 expression is absent in all checked human cancer cell lines (Fig. 7a). Furthermore, by collecting fresh normal and tumorous surgical samples from the same patient with CRC (Supplementary Table II), we found ZBP1 expression is significantly lower or absent in tumor compared to their matched noncancerous colonic tissues, while the expression of Casp-6, RIPK3 and MLKL were generally comparable in both normal and tumorous tissues (Fig. 7b). Thus, these data suggest that tumors may evade ZBP1-mediated cell death by downregulation of ZBP1 expression and ZBP1-drived PANoptosis is most likely activated by chemotherapy in noncancerous tissues.

To determine whether ZBP1-derived PANoptosis is activated by chemotherapy, we first examined dsRNA expression, as we showed it could be recognized by ZBP1 to trigger PANoptosis, in normal colonic tissues with/without oxaliplatin-based chemotherapy in CRC patients (Supplementary Table II). We found dsRNA expression was induced by oxaliplatin-based chemotherapy (Fig. 7c, d and Supplementary Fig. 7), and the dsRNA was most likely expressed in intestinal crypt cells and immune cells (Fig. 7c). We then performed fluorescent-immunohistochemistry assay in these samples by using dsRNA J2 and leukocyte common antigen CD45 antibodies. We found some of the dsRNA-positive cells were CD45 positive (Supplementary Fig. 8), indicating dsRNA was induced by chemotherapy in immune sentinel cells. Furthermore, the association between dsRNA and ZBP1 was detected by PLA assay in these normal colonic tissues with chemotherapy, indicating dsRNA can be sensed by ZBP1 in chemotherapy (Fig. 7e). To determine whether ZBP1-derived PANoptosome assembly was triggered by chemotherapy in cancer patients, we performed PLA assay in colonic tissues to examine the association between ZBP1 and RIPK3 or Casp-6, respectively. We detected the red fluorescent PLA signals in normal colonic tissues with chemotherapy, however, we found the PLA signals were absent in normal colonic tissues without chemotherapy (Fig. 7f and g). Thus, these data suggest that chemotherapy-induced dsRNA can be sensed by ZBP1 and trigger PANoptosis in noncancerous tissues in CRC patients.

## Discussion

Although DNA damage-induced apoptosis and necroptosis have been well characterized [33–35], the involvement of PANoptosis in DNA damage-induced cell death is not investigated. In this study, we found that ZBP1-derived PANoptosis was activated by DNA damage. Previous studies showed that ZBP1-derived PANoptosis was induced in infectious diseases [7, 9]. By binding viral-derived Z-RNA, ZBP1 triggers the assembly of the PANoptosome complex, which contains several key components in multiple PCD pathways, including RIPKs, NLRP3, ASC, Casp-6 and Casp-8 [7, 9]. In DNA damage, we demonstrated the assembled PANoptosome complex at least included ZBP1, RIPK3 and Casp6. Although RIPK1 is crucial for DNA damage-induced apoptosis and necroptosis [33], we found RIPK1 was not involved in the assembly of ZBP1-PANoptosome in response to DNA damage. This observation suggests that the assembly components in ZBP1-PANoptosome are varied under different stress conditions.

The two Zα-domains in ZBP1 have been shown to specifically bind nucleic acids in left-handed Z-conformation (Z-RNA or Z-DNA), which is essential for ZBP1 to trigger inflammatory cell death [40]. We found dsRNA was induced by DNA damage, and the binding of dsRNA to ZBP1 through its Zα-domain was crucial for DNA damage-induced PANoptosis. However, we could not detect the presence of Z-RNA in the cells by immunofluorescence staining with an anti-Z-NA Z22 antibody. It is believed that Z-RNA or Z-DNA duplexes were unstable and difficult to form under natural conditions in living cells [40]. However, previous studies showed that the Zα domain of an RNA editing enzyme ADAR1 could weakly bind right-handed RNA and promote its conformational transition into left-handed Z-form under near-physiological conditions in vitro [41, 42]. Indeed, the Zα domain of ZBP1 could also bind right-handed B-DNA and facilitate its transition into left-handed Z-DNA [43, 44]. Thus, although we could not detect the presence of Z-RNA in the cells with DNA damage, ZBP1 may bind right-handed dsRNA and facilitate its conformational transition into left-handed Z-form through its Zα domain. Or there may be other additional molecules that contribute to dsRNA recognition by ZBP1 in DNA damage. It would be worthwhile to investigate these possibilities in future studies.

ZBP1 has emerged as an important innate immune sensor of double-stranded nucleic acids in the Z-conformation [12]. Our data indicate that ERVs are transcriptionally upregulated by DNA damage and act as ligands for ZBP1 to trigger PANoptosis. Under normal conditions, the expression of ERVs is switched off epigenetically. Multiple processes, such as epigenetic aberrations, inflammation as well as infections can lead to the activation of ERVs [38]. It has been shown that 5-FU could activate ERVs to elicit inflammatory responses in hematopoietic stem cells [45]. However, to our knowledge, it has not yet been comprehensively demonstrated that ERVs can be transcriptionally upregulated by DNA damage. Our data indicate that DNA damage agents ETO and CDDP also activate ERVs. Therefore, one outstanding question is how ERVs are activated by DNA damage. It has been shown that 5-FU inhibited the expression of SETDB1 [45], a known epigenetic regulator in ERV silencing [46], which may lead to the activation of ERVs. Whether DNA damage agents share a similar mechanism to activate ERVs remains to be determined. And it will also be interesting to investigate whether the ADAR1 RNA editing enzyme is able to prevent ZBP1 activation and PANoptosis by disrupting dsRNA formation in DNA damage.

Our data reveal that ZBP1-mediated PANoptosis contributes to chemotherapy-induced toxicity and inflammation in mice. DNA damage agents, such as ETO, CDDP and 5-FU, are commonly used for cancer treatment. However, the optimal use of these chemotherapy agents is restricted by dose-limiting side effects, which may result from the damage of normal cells. Consistent with a previous study [39], we found ZBP1 expression was low or absent in tumor tissue, which suggests ZBP1-derived PANoptosis is most likely activated by chemotherapy in noncancerous tissues. Furthermore, we observed that dsRNA was induced by chemotherapy and can be sensed by ZBP1 in normal colonic tissues in CRC patients. Although we still need to gain more clinical evidence to comprehensively investigate the role of PANoptosis in chemotherapy-induced side effects, it would also be interesting to develop therapeutic approaches to mitigate side effects of chemotherapy by targeting ZBP1 in future studies.

In summary, our data reveal that ERVs are activated by DNA damage and can act as ligands for ZBP1 to trigger PANoptosis (Fig. 7h). This improved our understanding of the mechanistic basis in DNA damage-induced cell death. Furthermore, as ZBP1 expression is largely restricted to normal tissues in cancer patients, our findings suggest that ZBP1-derived PANoptosis contributes to chemotherapy-induced toxicity. These results, therefore, indicate that the inhibition of ZBP1-derived PANoptosis could be a potential therapeutic approach to alleviate chemotherapy-induced side effects and improve the quality of life in cancer patients.

## Supplementary information

Supplemental figure 1-Supplemental figure 8

Supplementary Table 1

Supplementary Table 2

uncropped WB figures

## Data Availability

The authors declare that all data supporting the findings of this study are available within the article and the Supplementary Information. All other data are available from the corresponding authors upon request.
