# Correction: Sensing of endogenous retroviruses-derived RNA by ZBP1 triggers PANoptosis in DNA damage and contributes to toxic side effects of chemotherapy

**DOI:** 10.1038/s41419-025-07676-z

**Published:** 2025-05-20

**Authors:** Fang Wang, Kaiying Li, Wensheng Wang, Hui Jiang, Jiangping He, Jin Cai, Wenqing Ren, Yaxing Zhao, Qianqian Song, Yuan He, Yanlei Ma, Xiaona Feng, Yue Liu, Jianqiang Yu, Siriporn Jitkaew, Dan Ma, Zhenyu Cai

**Affiliations:** 1 Tongji University Cancer Center, Shanghai Tenth People’s Hospital, School of Medicine, Tongji University, 200072 Shanghai, China; 2 Department of Biochemistry and Molecular Biology, School of Medicine, Tongji University, 200331 Shanghai, China; 3 Department of General Surgery, Xinqiao Hospital, Third Military Medical University, 400037 Chongqing, China; 4 Guangzhou National Laboratory, 510005 Guangzhou, China; 5 Department of Colorectal Surgery, Fudan University Shanghai Cancer Center, 200032 Shanghai, China; 6 College of Pharmacy, Ningxia Medical University, Yinchuan, 750004 Ningxia Hui Autonomous Region Yinchuan, China; 7 Center of Excellence for Cancer and Inflammation, Department of Clinical Chemistry, Faculty of Allied Health Sciences, Chulalongkorn University, Bangkok, 10330 Thailand; 8 State Key Laboratory of Cardiology and Medical Innovation Center, Shanghai East Hospital, School of Medicine, Tongji University, 200120 Shanghai, China

**Keywords:** Apoptosis, Necroptosis, Chemotherapy

Correction to: *Cell Death and Disease* 10.1038/s41419-024-07175-7, published online 27 October 2024

In this article the author name “Jiang Hui” is misspelled and should be read “Hui Jiang”; and the name “Jitkaew Siriporn” should be read “Siriporn Jitkaew”.

The original article has been corrected.

